# The ProteomeXchange consortium at 10 years: 2023 update

**DOI:** 10.1093/nar/gkac1040

**Published:** 2022-11-12

**Authors:** Eric W Deutsch, Nuno Bandeira, Yasset Perez-Riverol, Vagisha Sharma, Jeremy J Carver, Luis Mendoza, Deepti J Kundu, Shengbo Wang, Chakradhar Bandla, Selvakumar Kamatchinathan, Suresh Hewapathirana, Benjamin S Pullman, Julie Wertz, Zhi Sun, Shin Kawano, Shujiro Okuda, Yu Watanabe, Brendan MacLean, Michael J MacCoss, Yunping Zhu, Yasushi Ishihama, Juan Antonio Vizcaíno

**Affiliations:** Institute for Systems Biology, Seattle WA 98109, USA; Center for Computational Mass Spectrometry, University of California, San Diego (UCSD), La Jolla, CA 92093, USA; Dept. Computer Science and Engineering, University of California, San Diego (UCSD), La Jolla, CA 92093, USA; Skaggs School of Pharmacy and Pharmaceutical Sciences, University of California, San Diego (UCSD), La Jolla, CA 92093, USA; European Molecular Biology Laboratory, European Bioinformatics Institute (EMBL-EBI), Wellcome Trust Genome Campus, Hinxton, Cambridge, CB10 1SD, UK; University of Washington, Seattle, WA 98195, USA; Center for Computational Mass Spectrometry, University of California, San Diego (UCSD), La Jolla, CA 92093, USA; Dept. Computer Science and Engineering, University of California, San Diego (UCSD), La Jolla, CA 92093, USA; Skaggs School of Pharmacy and Pharmaceutical Sciences, University of California, San Diego (UCSD), La Jolla, CA 92093, USA; Institute for Systems Biology, Seattle WA 98109, USA; European Molecular Biology Laboratory, European Bioinformatics Institute (EMBL-EBI), Wellcome Trust Genome Campus, Hinxton, Cambridge, CB10 1SD, UK; European Molecular Biology Laboratory, European Bioinformatics Institute (EMBL-EBI), Wellcome Trust Genome Campus, Hinxton, Cambridge, CB10 1SD, UK; European Molecular Biology Laboratory, European Bioinformatics Institute (EMBL-EBI), Wellcome Trust Genome Campus, Hinxton, Cambridge, CB10 1SD, UK; European Molecular Biology Laboratory, European Bioinformatics Institute (EMBL-EBI), Wellcome Trust Genome Campus, Hinxton, Cambridge, CB10 1SD, UK; European Molecular Biology Laboratory, European Bioinformatics Institute (EMBL-EBI), Wellcome Trust Genome Campus, Hinxton, Cambridge, CB10 1SD, UK; Center for Computational Mass Spectrometry, University of California, San Diego (UCSD), La Jolla, CA 92093, USA; Dept. Computer Science and Engineering, University of California, San Diego (UCSD), La Jolla, CA 92093, USA; Skaggs School of Pharmacy and Pharmaceutical Sciences, University of California, San Diego (UCSD), La Jolla, CA 92093, USA; Center for Computational Mass Spectrometry, University of California, San Diego (UCSD), La Jolla, CA 92093, USA; Dept. Computer Science and Engineering, University of California, San Diego (UCSD), La Jolla, CA 92093, USA; Skaggs School of Pharmacy and Pharmaceutical Sciences, University of California, San Diego (UCSD), La Jolla, CA 92093, USA; Institute for Systems Biology, Seattle WA 98109, USA; Faculty of Contemporary Society, Toyama University of International Studies, Toyama 930-1292, Japan; Database Center for Life Science (DBCLS), Joint Support-Center for Data Science Research, Research Organization of Information and Systems, Chiba 277-0871, Japan; School of Frontier Engineering, Kitasato University, Sagamihara 252-0373, Japan; Niigata University Graduate School of Medical and Dental Sciences, Niigata 951-8510, Japan; Niigata University Graduate School of Medical and Dental Sciences, Niigata 951-8510, Japan; University of Washington, Seattle, WA 98195, USA; University of Washington, Seattle, WA 98195, USA; Beijing Proteome Research Center, National Center for Protein Sciences, Beijing Institute of Lifeomics, Beijing 102206, China; Graduate School of Pharmaceutical Sciences, Kyoto University, Kyoto 606-8501, Japan; European Molecular Biology Laboratory, European Bioinformatics Institute (EMBL-EBI), Wellcome Trust Genome Campus, Hinxton, Cambridge, CB10 1SD, UK

## Abstract

Mass spectrometry (MS) is by far the most used experimental approach in high-throughput proteomics. The ProteomeXchange (PX) consortium of proteomics resources (http://www.proteomexchange.org) was originally set up to standardize data submission and dissemination of public MS proteomics data. It is now 10 years since the initial data workflow was implemented. In this manuscript, we describe the main developments in PX since the previous update manuscript in *Nucleic Acids Research* was published in 2020. The six members of the Consortium are PRIDE, PeptideAtlas (including PASSEL), MassIVE, jPOST, iProX and Panorama Public. We report the current data submission statistics, showcasing that the number of datasets submitted to PX resources has continued to increase every year. As of June 2022, more than 34 233 datasets had been submitted to PX resources, and from those, 20 062 (58.6%) just in the last three years. We also report the development of the Universal Spectrum Identifiers and the improvements in capturing the experimental metadata annotations. In parallel, we highlight that data re-use activities of public datasets continue to increase, enabling connections between PX resources and other popular bioinformatics resources, novel research and also new data resources. Finally, we summarise the current state-of-the-art in data management practices for sensitive human (clinical) proteomics data.

## INTRODUCTION

Mass spectrometry (MS)-based proteomics approaches are increasingly used as a highly-valuable tool in biomedical research. Key applications of proteomics are the study of baseline or differential protein expression, characterization of protein primary structures and their post-translational modifications (PTMs, e.g. phosphorylation), the elucidation of protein structures and the study of protein-protein interactions, among others. Proteomics often complements other omics technologies such as genomics, transcriptomics, lipidomics, glycomics and metabolomics.

The ProteomeXchange (PX) consortium of proteomics resources (1–3) (http://www.proteomexchange.org) aims to standardize data submission and dissemination of public MS proteomics data worldwide. PX resources are committed to comply with the FAIR (Findable, Accessible, Interoperable, Re-usable) principles (4) for biological data, support reproducible research and represent the state-of-the-art in proteomics with regards to open data practices. The perceived reliability of PX resources has enabled an unprecedented increase in the amount of proteomics data in the public domain.

The first implementation of the PX consortium data workflow took place in 2012. At the time, it involved only two resources: the PRIDE database (5) (European Bioinformatics Institute, EMBL-EBI, Hinxton, UK) and the PASSEL (6) resource within PeptideAtlas (Institute for Systems Biology, Seattle, USA). Additionally, PeptideAtlas (7) participated by reanalysing public submitted datasets. Four additional resources have joined PX over the years, which are listed next in chronological order: MassIVE (University of California San Diego, USA, in 2014), jPOST (8) (the jPOST project, Japan, in 2016), iProX (9) (National Center for Protein Sciences, Beijing, China, in 2017), and Panorama Public (10) (University of Washington, Seattle, USA, in 2018). A common portal called ProteomeCentral (http://proteomecentral.proteomexchange.org) provides search capabilities for public datasets in all participating PX resources, since it contains a summary of metadata information for each public dataset.

As a key point, the work of PX is very closely aligned with the activities of the Proteomics Standards Initiative (PSI, https://www.psidev.info/), the organization which develops community-based open data standards in the field (11,12). PX resources support and implement the main MS related PSI open data formats and the relevant controlled vocabularies. Additionally, they develop and maintain several open-source parser libraries and tools to support these data standards, e.g. (13).

During these first 10 years, thanks to the perceived reliability of PX resources and the data policies established by scientific journals and funding agencies, and also because of the fact that public data sharing is now widely considered to be a good scientific practice, the proteomics field has embraced open data practices. This has been a tremendously positive development for the field for multiple reasons. Foremost is that multiple types of data re-use activities are increasingly contributing to the field, as is described in detail below.

Here we provide an update of the activities of the PX consortium and its individual resources since the previous update paper was published in *Nucleic Acids Research* (NAR) three years ago (5). We also describe updated submission statistics to demonstrate the continuous growth of proteomics datasets in the public domain and the wide adoption of PX. As a key point we highlight data re-use activities in the context of the PX resources but also by third parties, and discuss future developments. Please see the latest update manuscripts of the individual PX resources for more comprehensive information about each of them separately (5,9,10,14).

## CURRENT PX DATA WORKFLOW AND IMPLEMENTATION OF PSI DATA STANDARDS

PRIDE, MassIVE, jPOST and iProX are *universal* archival resources, while PASSEL and Panorama Public are *focused* resources aimed at targeted proteomics approaches. All PX resources store MS proteomics data providing private access for reviewers and journal editors during the manuscript review process. See Table 1 for information about how to access each resource. Additionally, Table 2 provides a summary of the main functionality offered by the PX resources. PX dataset (PXD) identifiers are persistent and unique, and are used as the main dataset identifier for all originally submitted datasets compliant with PX requirements (https://registry.identifiers.org/registry/px). RPXD identifiers are issued in some cases for reanalysed datasets. Additionally, some PX resources have their own identifiers for datasets, that can also be used in parallel to the PXD identifiers. Furthermore, Digital Object Identifiers (DOIs) can also be issued for ‘Complete’ submissions (see below for more details about submission types) and PXD identifiers are resolved by the identifier resolution services identifiers.org (15) and Bioregistry (16). In terms of data license, all PX resources moved to a default Creative Commons CC0 license as the basis in 2020. However, Panorama Public and iProX assign a CC-BY license, which requires attribution, as the default, with CC0 available as an option to data submitters.

**Table 1.** Overview information of the current PX resources

Resource name	Institution, country	URL	Contact	Documentation pages
PRIDE	European Bioinformatics Institute (EMBL-EBI), Cambridge, UK	http://www.ebi.ac.uk/pride	pride-support@ebi.ac.uk	https://www.ebi.ac.uk/pride/markdownpage/submitdatapage
PeptideAtlas	Institute for Systems Biology, Seattle, WA, USA	http://www.peptideatlas.org/	http://www.peptideatlas.org/feedback.php	http://www.peptideatlas.org/software.php
PASSEL	Institute for Systems Biology, Seattle, WA, USA	http://www.peptideatlas.org/passel/	http://www.peptideatlas.org/feedback.php	http://www.peptideatlas.org/passel/
MassIVE	University of California, San Diego, CA, USA	https://massive.ucsd.edu/	ccms-web@cs.ucsd.edu	https://ccms-ucsd.github.io/MassIVEDocumentation/
jPOST	The jPOST project, Japan	https://jpostdb.org/	https://repository.jpostdb.org/contact	https://repository.jpostdb.org/help
iProX	National Center for Protein Sciences, Beijing, China	https://www.iprox.org/	iprox@iprox.org	https://www.iprox.org/page/helpEn.html
Panorama Public	University of Washington, Seattle, WA, USA	https://panoramaweb.org/public.url	panorama@proteinms.net	https://panoramaweb.org/public_docs.url

**Table 2.** Main functionality offered by the PX resources

Functionality	PRIDE	PASSEL	MassIVE	jPOST	iProX	Panorama Public	PeptideAtlas
**Types of data access**							
Web interface	Yes	Yes	Yes	Yes	Yes	Yes	Yes
Application Programming Interface	Yes	Yes	Yes	Yes	Yes	Yes	Yes
Protocol for file transfer (download/ upload)	FTP, Aspera	FTP	FTP	FTP, HTTPS, TripleStore	HTTP, Aspera	WebDAV, HTTPS	FTP
Reviewer private access	File download	File download	File download, web interface	File download	File download, web interface	File download, web interface	N/A
**General functionality/web visualization**							
Dataset centric view	Yes	Yes	Yes	Yes	Yes	Yes	Yes
Protein centric view across resource	No	Yes	Yes	No	Yes	Yes	Yes
Annotated mass spectra	Yes	Yes	Yes	Yes	Yes	Yes	Yes
USIs	Yes	Yes	Yes	Yes	Yes	No	Yes
Chromatograms	No	Yes	Yes	No	No	Yes	No
**Data license**	CC0	CC0	CC0	CC0	CC-BY (default) CC0 (optional)	CC-BY (default) CC0 (optional)	CC0

**Abbreviations:** API: Application Programming Interface; EGA: European Genotype-phenome Archive; DDA: Data Dependent Acquisition; DIA: Data Independent Acquisition; DL: Deep Learning; DOI: Digital Object Identifier; FAIR: Findable, Accessible, Interoperable, Re-usable; GDPR: General Data Protection Regulation; HPP: Human Proteome Project; HUPO: Human Proteome Organization; IDF: Investigation Description Format; JGA: Japanese Genotype-phenotype Archive; JPDM: Journal of Proteome Data and Methods; ML: Machine Learning; MS: Mass Spectrometry; OmicsDI: Omics Discovery Index; ORF: Open Reading Frame; PDB: Protein Data Bank; PSI: Proteomics Standards Initiative; PTM: Post-Translational Modification; PX: ProteomeXchange; RSS: Rich Site Summary; SDRF: Sample and Data Relationship Format; UCSC: University of California, Santa Cruz; UniProtKB: UniProt KnowledgeBase; USI: Universal Spectrum Identifier.

The overall data workflow has not changed in the last three years. First, researchers submit data to one of the PX data resources. Second, the data remains private during the manuscript review process. Third, once the accepted manuscript is published, the corresponding dataset(s) are made publicly available and disseminated to ProteomeCentral. At that point, the datasets become available to everyone in the community and can be re-used. There are two data submission workflows, called ‘Complete’ and ‘Partial’. For both submission types, a set of common experimental metadata at the level of each dataset must be included (encoded in the shared PX XML format used by ProteomeCentral, http://proteomecentral.proteomexchange.org/schemas/proteomeXchange-1.4.0.xsd), together with the raw mass spectra and the processed results (identification and/or quantification data).

The key difference between both submission types is that in the case of a ‘Complete’ dataset, it is required that the receiving PX resource is able to parse, process and directly connect all individual results with the submitted MS data, making data visualization possible. This can only be usually done if the processed results are available in supported PSI open standard data formats. PX resources fully support the main open PSI data standards for MS, namely mzML (for MS data) (17), mzIdentML (18) and mzTab (tab-delimited file for peptide and protein identification and quantification) (19). Additionally, there are other open formats produced by the Skyline software (20) that are supported by Panorama Public for ‘Complete’ submissions.

In contrast, ‘Partial’ datasets contain processed result files that are not in open standard formats that can be parsed and thus ingested by the receiving repository. Any analysis output file is then allowed. This mode is required to support datasets analysed using dozens of analysis tools not supporting open data standards and generated coming from so many different experimental approaches. Such ‘Partial’ datasets can be downloaded and re-used if the end user has suitable software to parse or visualize the files. Or more often, the data may be reprocessed and reinterpreted using the raw data as the basis.

In the context of the FAIR data principles, all resources in PX apart from PanoramaPublic now support PSI’s Universal Spectrum Identifiers (USIs) for mass spectra (21), formalized in 2021. USIs provide a standardized mechanism for encoding a virtual path to any mass spectrum contained in datasets deposited to PX (https://registry.identifiers.org/registry/mzspec). Therefore, USIs enable greater transparency of spectral evidence making it more ‘FAIR’. ProteomeCentral implements a single endpoint at http://proteomecentral.proteomexchange.org/usi/ that reaches out to all participating partners to fetch spectra for a provided USI if available at any resource. Spectrum interpretations are also supported as part of the USI using the ProForma 2.0 (22) notation for peptidoforms. In addition to PX resources supporting the original submitted spectra interpretations, a subset of them (e.g. ProteomeCentral, MassIVE and PeptideAtlas) allow users to experiment with alternative interpretations of the same spectra, thus facilitating interactive assessment of the quality of spectrum identifications. For instance, MassIVE USI query tools also allow users to consider additional information to support or dispute the reported identifications: (i) by enabling searching for alternative identifications for the same USI spectrum (possibly from datasets reanalyses), and (ii) by enabling looking for reference spectra for the same USI peptide (e.g. from reference spectral libraries such as MassIVE-KB (23)).

## IMPROVEMENTS IN PROVISION OF EXPERIMENTAL METADATA ENABLES DATA REANALYSIS

An additional recent development in the PX data workflow is the development of the file format MAGE-TAB-Proteomics to enable an improved metadata annotation of PX datasets (24). The lack of appropriate structured metadata at the sample level, including the experimental design, can prevent a more streamlined re-use of the available public datasets in PX resources, especially in the case of reanalyses of quantitative proteomics datasets. The MAGE-TAB-Proteomics format is an extension of the original MAGE-TAB format used in transcriptomics and has two main components: the Investigation Description Format (IDF) and the Sample and Data Relationship Format (SDRF-Proteomics). First, the IDF contains the general description of the study (PX users do not need to provide it because the file can be generated by the resources based on the current information provided by the submitters). Second, the SDRF-Proteomics format includes the representation of the experimental design, and the mappings between the samples in the experiment and the raw files. SDRF-Proteomics is a tab-delimited format where each column is a property of the sample or the data file (https://github.com/bigbio/proteomics-metadata-standard). SDRF-Proteomics files can now be created using a spreadsheet software (e.g. Excel^®^) and be added by submitters to each submitted dataset to PRIDE. As of September 2022, approximately 450 PRIDE datasets had associated SDRF-Proteomics files, which were provided either by the submitters or by third parties that reannotated the datasets, see the list of public datasets at: https://www.ebi.ac.uk/pride/archive?keyword=sdrf.tsv).

Before the development of MAGE-TAB-Proteomics, MassIVE introduced the MassIVE.quant resource (25) (in collaboration with Northeastern University) for the sharing of quantitative proteomics datasets, metadata and reanalyses. Compatible with all major MS data acquisition types and computational analysis tools, MassIVE.quant systematically stores the raw data, the experimental design, the scripts (or descriptions) of every step of the quantitative analysis workflow, and the intermediate input and output files. MassIVE.quant annotation of quantitative datasets now covers 128 915 spectrum files in public datasets corresponding to 33 314 samples in thousands of study groups.

jPOST have also focused as well in developing workflows to reanalyze submitted data in a unified procedure. To enable that, several methods had to be implemented to improve the collection of experimental metadata. In addition to extracting the metadata from scientific articles by manual curation, a data journal called *Journal of Proteome Data and Methods* (JPDM, https://www.jhupo.org/jpdm/) was launched. This journal gives incentives for data contributors to provide detailed metadata in the form of articles [https://doi.org/10.14889/jpdm.2019.0001]. Based on the metadata collected in this way, more than 100 datasets have been reanalysed, assigned RPXD identifiers, and published from jPOSTrepo. Reanalysed datasets have also been made available from jPOSTdb (14), which is equipped with a protein viewer. The jPOST team will continue to reanalyse submitted datasets based on the collected available metadata. The current plan is also to develop a mechanism to automatically collect metadata from papers through machine learning (ML), based on the relationship between the submitted data and the metadata collected.

## DATA SUBMISSION AND DATA ACCESS STATISTICS

**Figure 1.**
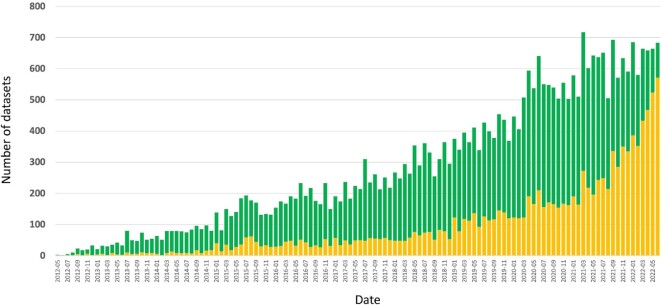
Number of submitted datasets per month to PX resources, ranging from May 2012 to June 2022. Publicly released datasets are colored in green, unreleased datasets are colored in yellow.

As of the end of June 2022, a total of 34 233 datasets had been submitted to PX resources. Of those, 22 675 datasets (66.2%) were already publicly available, whereas the rest were still unreleased (11 558 datasets, 33.8%). The number of submitted datasets has increased year after year, a trend that has not stopped yet (Figure 1). Since the previous PX update paper (3), 20 064 datasets have been submitted to PX resources, meaning that 58.6% of PX datasets were submitted within just the last three years. This again showcases the very significant increase of proteomics datasets in the public domain. During 2021 alone, a record number of 7333 datasets were submitted to PX resources (611 datasets per month on average). During the first 6 months of 2022, this number has been 3935 datasets.

In terms of distribution of datasets submitted across individual PX resources, 28 473 datasets (83.2%), had been submitted to PRIDE, followed by MassIVE (2360 datasets, 6.9%), iProX (1893 datasets, 5.5%), jPOST (1086 datasets, 3.2%), Panorama Public (277 datasets, 0.81%) and PeptideAtlas/PASSEL (144 datasets, 0.42%). As of August 2022, datasets came from at least 76 different countries, demonstrating further the global reach of PX. Additionally, datasets came from more than 3818 taxonomy IDs. As of the end of December 2021, the combined file size of all PX resources was ∼2.63 petabytes. Detailed download statistics for all PX resources during 2019, 2020 and 2021 can be accessed at Supplementary Table S1.

## DATA RE-USE ACTIVITIES

**Figure 2.**
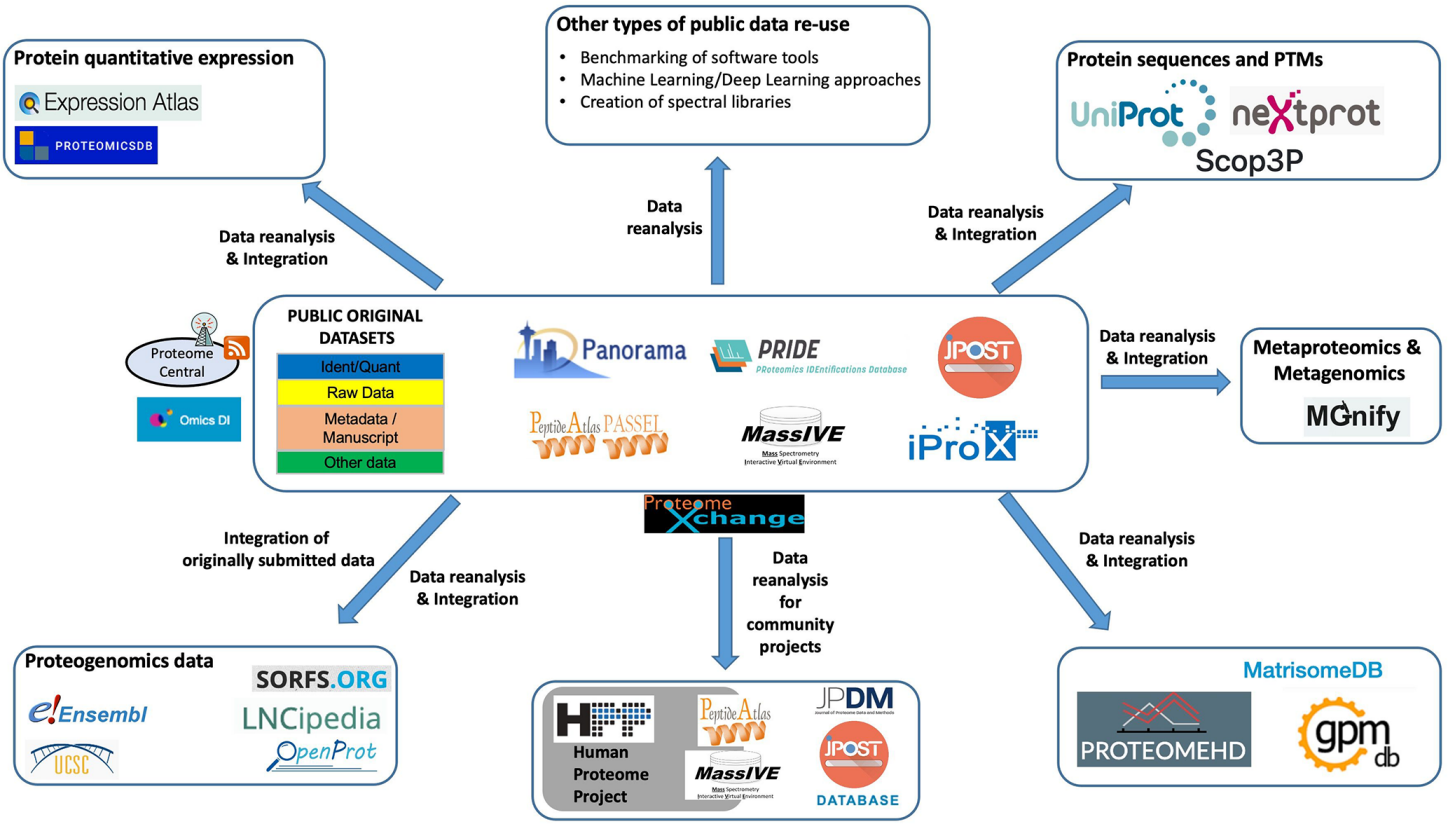
Overview figure including the current PX resources and the main efforts devoted to data re-use of public proteomics datasets. Different data types are listed, including protein quantitative expression, integration of genomics and proteomics data (proteogenomics), including metagenomics and metaproteomics, peptide and protein sequences, and PTMs. For each data type, the corresponding data resources where these data can be accessed are highlighted. Additional data re-use activities are also indicated, e.g. the efforts in the context of the Human Proteome Project, software benchmarking, machine learning approaches and the creation of spectral libraries. Finally, other bioinformatics resources re-using proteomics data are also indicated (ProteomeHD, MatrisomeDB and GPMDB).

Enabled by PX resources, data re-use activities, including the reanalysis of public proteomics datasets, are increasing dramatically, as summarized in Figure 2. Systematic reprocessing of public PX datasets by PX resources is a core activity towards making proteomics data more FAIR: Findable (e.g. indexing standardized search results), Accessible (e.g. online data exploration tools that do not require full dataset downloads), Re-usable (e.g. results reported in standard open formats) and Interoperable (e.g. search results reported using standard protein identifiers). At the level of the PX data resources, many of these data re-use efforts aim to make proteomics data more accessible to life scientists, especially to those non-experts in proteomics. These activities involve different data types generated from proteomics experiments:

- Peptide and protein sequences and PTM data. MassIVE has developed freely several accessible open-source workflows for systematic dataset reanalysis including e.g. the MODa open-modification search for the detection of unexpected modifications (26). Altogether, MassIVE has reanalysed over 2.2 billion mass spectra from 392 datasets to derive over 1.1 billion new peptide identifications. To facilitate data re-use, MassIVE provides automated workflows to convert submitted MS raw data into mzML and has already used these to release billions of spectra in tens of thousands of converted raw files. Repository-scale integration of proteomics data requires specialized workflows to avoid accumulation of false discoveries across datasets. MassIVE addressed this problem by developing the MassIVE-KB workflow (23) for the construction of spectral libraries with globally controlled false discovery rates (FDR) at the spectrum, peptide and protein levels. The current release of the human MassIVE-KB spectral library (https://massive.ucsd.edu/ProteoSAFe/static/massive-kb-libraries.jsp) was constructed from 326 million identifications derived from over 1.2 billion spectra, with the resulting library containing over 6 million reference spectra for 19 855 (>97% of all) canonical human proteins.

Using similar workflows, PeptideAtlas has also released species-specific builds based on PX data, including builds for human, *Arabidopsis* (27), yeast, rohu (28) and *Pseudomonas aeruginosa* (29). SWATHAtlas (http://www.swathatlas.org/) provides spectral libraries suitable for DIA (Data Independent Acquisition) analysis for over a dozen different species, all validated for quality with DIALib-QC (30). These efforts by PX resources were then integrated with the Human Proteome Organization (HUPO) flagship initiative on the Human Proteome Project (HPP) to constitute the largest-ever community-scale data reanalysis project to construct the human proteome blueprint (31) establishing the protein-level existence of gene products for ∼90% of the human genome—a flagship achievement of the whole proteomics community that would not have been possible without the data sharing infrastructure provided by PX resources.

jPOST also reanalyzes human, mouse, *Escherichia coli*, SARS-CoV-2, and other datasets from submitted raw data and provides them via jPOSTdb (14). It is important to highlight that PX resources already integrate peptide and protein sequence data into protein knowledge-bases such as UniProtKB (UniProt KnowledgeBase) (32) and neXtProt (33). Additionally, we are working in developing data pipelines, file formats and guidelines to provide PTM data (starting with phosphorylation) to UniProtKB, in the context of the ‘PTMeXchange’ project. So far, we have devoted efforts to the benchmarking of a method to accurately report PTMs with a global false localisation rate (34) and have started working in the reanalysis and integration of phospho-enriched datasets coming from rice, *Plasmodium falciparum* and mouse. Future work will be devoted to human PTM data, including other protein modifications as well. Outside the members of the PX consortium, other bioinformatics resources for providing PTM reanalyses of PX datasets have also been started in recent years, including Scop3P (35). Additionally, GPMDB (36) has been providing to the community re-analysed peptide, protein and PTM identification data for >15 years.

- Data coming from proteogenomics approaches (also including immunopeptidomics and metaproteomics approaches). On one hand, it should be highlighted that peptide sequence data can be integrated in resources such as Ensembl (37), Ensembl Genomes or the UCSC genome browser (38) by using proteomics data ‘hubs’. Additionally, public PX datasets can be reanalysed using sequence databases constructed by e.g. using genomics, transcriptomics or Ribo-seq data, among other DNA/RNA sequencing approaches. The original application of these proteogenomics approaches is to improve genome annotation efforts. Some recent efforts involving PeptideAtlas involve the reanalysis of some datasets to provide experimental evidence of the expression of ORFs (Open Reading Frames) detected using Ribo-seq (39).

Outside the work of PX partners, some bioinformatics resources have been set up to provide proteomics evidence for some genomics events, e.g. LNCipedia (40) (for long-non-coding RNAs), sORF.org (41) (for short ORFs) and OpenProt (42) (proteogenomics resource supporting a polycistronic annotation model for eukaryotic genomes). Additionally, in a wider context of proteogenomics approaches, some pilot work has been performed by PRIDE to link and integrate metaproteomics datasets with the corresponding metagenomics and metatranscriptomics data in the EMBL-EBI’s resource MGnify (43). Furthermore, the amount of inmunopeptidomics datasets in the public domain is also increasing. The resource SysteMHC Atlas (44) was set-up to represent this data type, linking to the original public datasets in PX resources. The resource is at present no longer available in the public domain, although there are ongoing plans to re-develop it in a new infrastructure.

- Protein quantitative expression information. There are different efforts to provide consistently reanalysed quantitative proteomics data. PRIDE is integrating protein expression information in the EMBL-EBI’s resource Expression Atlas (45), enabling the access and visualization of gene and protein expression (abundance) data in the same web interface. Different groups of datasets have been reanalysed and integrated so far, mainly Data Dependent Acquisition (DDA) data coming from cell lines and tumour tissues (46), human (47), mouse and rat tissues (48), and also a pilot study involving DIA datasets coming from different origins (49). Expression Atlas could also provide a future way to integrate single-cell proteomics data *via* the single-cell Expression Atlas.

Also in the context of quantitative proteomics, as mentioned above, MassIVE.quant (25) is a data resource for reproducible quantitative MS-based proteomics. As of September 2022, MassIVE.quant supports the dissemination of 209 quantitative reanalyses including the metadata, provenance records and all intermediate files required for reproducing the statistical analyses of 605 496 protein measurements resulting in 114 262 statistically-significant differential abundance events.

Outside the PX consortium, proteomicsDB (50) is a resource providing protein and gene expression data coming from human, mouse, *Arabidopsis* and rice at present. Many of the datasets used in proteomicsDB are generated locally at the group at the Technical University of Munich, but others are taken from PX resources.

Additionally, new data resources that re-used public PX datasets have also been set up in recent years, such as MatrisomeDB (51), providing an updated view of the human and mouse extracellular matrix, and ProteomeHD (52), a resource providing information about co-expressed proteins, among others.

In the community as a whole, public datasets are being re-used for other purposes in addition to the topics cited above. Benchmarking of software remains one of the most popular types of data re-use. Additionally, one key use case is the re-use of datasets in the application of popular ‘big data’ approaches involving proteomics data, such as ML and deep learning (DL) studies. Most studies make use of public datasets (e.g. for training purposes) in the development of ML/DL approaches, including e.g. the prediction of protein digestion, peptide retention time, peptide fragmentation, collision cross-section for ion mobility and/or improvements in algorithms for peptide and protein identification and quantification (for a recent review, see (53)), among other applications. In this context, PRIDE participated in a study using ML approaches to create a functional score for human phosphosites (54), where 112 human phospho-enriched datasets were reanalysed.

In order to facilitate access for data re-use purposes, public datasets in PX resources are also accessible through the OmicsDI (Omics Discovery Index) portal (http://www.omicsdi.org) (55). Among other functionality available, OmicsDI enables to link where possible, proteomics datasets included in multi-omics studies to the corresponding public datasets coming from other omics approaches (e.g. studies where both proteomics and transcriptomics datasets have been generated).

## SUPPORT FOR SENSITIVE HUMAN PROTEOMICS DATASETS

Led by some of the PX partners and members of the ELIXIR Proteomics community in Europe (https://elixir-europe.org/communities/proteomics), a community-driven white paper was published last year describing the current state of affairs in data management practices for sensitive human (clinical) proteomics datasets (56). Addressing ethical issues for genomics and transcriptomics data has led to data management processes to control who may access the data in so-called ‘controlled access’ repositories. This means that scientists wanting to obtain access to certain datasets need to write an application, which then must be approved by e.g. a Data Access Committee. Resources supporting the storage and dissemination of controlled access DNA/RNA sequencing datasets include the EGA (European Genome-phenome Archive) (57), dbGAP (USA) (58) and the Japanese Genotype-phenotype Archive (JGA) (59).

Currently all data in PX is open and publicly accessible. The necessity of similar controlled access options for proteomics data depends first of all, on whether these data can potentially be used to identify research participants. In proteomics, although a small number of studies in this topic have been published, especially in the context of forensic studies, more research is required for answering this question for the different experimental workflows and proteomics data types (56). In addition to issues related to the identifiability of individuals, controlled access to proteomics data may become necessary because of requirements related to patient consent and/or due to personal data regulations like GDPR (General Data Protection Regulation) in Europe, or any other relevant legislation.

The current policy in PX (as it is the case for other open resources storing other types of omics data) is that the submitter is responsible for guaranteeing that the data can be hosted legally by the corresponding PX resource they are submitting to. We anticipate that there will be an increasing number of sensitive (clinical) human datasets that cannot be made available *via* a fully open PX resource due to ethical-related issues (60). We recommend to users that if there are any potential legal issues of this type, they should submit their data to an alternative repository outside PX. However, existing controlled access resources such as those mentioned above (EGA, dbGaP and JGA) are not ideal for proteomics datasets. Their data model is based on the Sequence Read Archive data model, which is tailored for sequencing-based assays and cannot appropriately represent proteomics datasets.

To address this problem, some of the PX members will be working in developing a tailored infrastructure for storing and accessing sensitive human proteomics data, and in parallel, in all the related data policies. At the time of writing, PRIDE has started the design of such system, in collaboration with the EGA team at EMBL-EBI. MassIVE has also designed a platform for controlled access datasets but implementation is currently contingent on pending support for further developments. In China, the ‘Regulations of the People's Republic of China on the Management of Human Genetic Resources’ were implemented on 1 July 2019. Since their formal promulgation, the Beijing Proteome Research Center's Genetic Information Preservation Database (dbPDPM) and the Chinese National Population Health Data Center have been authorized to collect, preserve, utilize and provide the Chinese human genetics resource. It is planned that dbPDPM, which will be an extension of iProX to support multi-omics data, and is planned to be launched at the end of 2022.

## DISCUSSION AND FUTURE PLANS

PX continues to support the open data culture in the proteomics field by promoting and enabling the sharing of proteomics data. An increasing number of scientific journals (including the main proteomics ones) and funding agencies are mandating the submission of the generated datasets accompanying the submitted manuscripts. This is of course one of the main reasons behind the continuous growth in submitted datasets.

PX resources continue to evolve in parallel to the needs of the field. In the context of data archiving activities, in addition to the already-covered topic of sensitive proteomics datasets, improved support will be provided for structural proteomics datasets, including linking submissions of different structural data to the Protein Data Bank (PDB) (61). Support for DIA approaches would ideally need to be improved in different ways, since the original PX data submission workflows were developed having DDA approaches in mind. We plan to provide a better support for the submission of spectral libraries in DIA datasets (which is currently optional), by making deposition mandatory, and by promoting the use of PSI’s mzSpecLib (https://github.com/HUPO-PSI/mzSpecLib) open data standard for spectral libraries, which is currently under development (62).

In the context of data re-use activities, we plan to continue with the activities mentioned above, for different purposes (e.g. quantitative protein expression, proteogenomics, peptide and protein sequence data and PTMs, creation of spectral libraries, etc). We think these data re-use and data integration efforts (as part of the existing wider trend in the field) are key for making proteomics data vastly more accessible and re-usable in the life sciences.

Another topic that we will follow closely is the further development of non-MS-based proteomics technologies such as the use of affinity reagents (e.g. SomaLogic^®^ and Olink^®^ assays). Tailored repositories for these data types are still lacking and may be needed. One possibility in the medium term is that future extensions of the existing PX resources, together with guidelines for metadata and dedicated software tools, will have to be developed to support these non-MS experiments. However, it is important to highlight that a large proportion of the studies generated to date from non-MS approaches are generated from human clinical samples, and then the data may be considered sensitive so that controlled access mechanisms may have to apply.

It is important to note that the consortium remains open to accept new members, provided that they adhere to the consortium requirements set out in the updated ProteomeXchange Membership Agreement (http://www.proteomexchange.org/pxcollaborativeagreement.pdf). For regular announcements of all the new publicly available datasets, users can follow our Twitter account (@proteomexchange) or subscribe to the following Rich Site Summary (RSS) feed (https://groups.google.com/forum/feed/proteomexchange/msgs/rss_v2_0.xml).

## DATA AVAILABILITY

The PX webpage is available at http://www.proteomexchange.org. No new data were generated or analysed in support of this research.

## Supplementary Material

gkac1040_Supplemental_File

## References

[1] Vizcaino J.A. , Deutsch E.W., Wang R., Csordas A., Reisinger F., Rios D., Dianes J.A., Sun Z., Farrah T., Bandeira N. et al. ProteomeXchange provides globally coordinated proteomics data submission and dissemination. Nat. Biotechnol. 2014; 32:223–226. 24727771 10.1038/nbt.2839 PMC3986813

[2] Deutsch E.W. , Csordas A., Sun Z., Jarnuczak A., Perez-Riverol Y., Ternent T., Campbell D.S., Bernal-Llinares M., Okuda S., Kawano S. et al. The proteomexchange consortium in 2017: supporting the cultural change in proteomics public data deposition. Nucleic Acids Res. 2017; 45:D1100–D1106. 27924013 10.1093/nar/gkw936 PMC5210636

[3] Deutsch E.W. , Bandeira N., Sharma V., Perez-Riverol Y., Carver J.J., Kundu D.J., Garcia-Seisdedos D., Jarnuczak A.F., Hewapathirana S., Pullman B.S. et al. The proteomexchange consortium in 2020: enabling ‘big data’ approaches in proteomics. Nucleic Acids Res. 2020; 48:D1145–D1152. 31686107 10.1093/nar/gkz984 PMC7145525

[4] Wilkinson M.D. , Dumontier M., Aalbersberg I.J., Appleton G., Axton M., Baak A., Blomberg N., Boiten J.W., da Silva Santos L.B., Bourne P.E. et al. The FAIR guiding principles for scientific data management and stewardship. Sci. Data. 2016; 3:160018. 26978244 10.1038/sdata.2016.18 PMC4792175

[5] Perez-Riverol Y. , Bai J., Bandla C., Garcia-Seisdedos D., Hewapathirana S., Kamatchinathan S., Kundu D.J., Prakash A., Frericks-Zipper A., Eisenacher M. et al. The PRIDE database resources in 2022: a hub for mass spectrometry-based proteomics evidences. Nucleic Acids Res. 2022; 50:D543–D552. 34723319 10.1093/nar/gkab1038 PMC8728295

[6] Farrah T. , Deutsch E.W., Kreisberg R., Sun Z., Campbell D.S., Mendoza L., Kusebauch U., Brusniak M.Y., Huttenhain R., Schiess R. et al. PASSEL: the peptideatlas SRMexperiment library. Proteomics. 2012; 12:1170–1175. 22318887 10.1002/pmic.201100515 PMC3832291

[7] Deutsch E.W. , Lam H., Aebersold R. PeptideAtlas: a resource for target selection for emerging targeted proteomics workflows. EMBO Rep. 2008; 9:429–434. 18451766 10.1038/embor.2008.56 PMC2373374

[8] Okuda S. , Watanabe Y., Moriya Y., Kawano S., Yamamoto T., Matsumoto M., Takami T., Kobayashi D., Araki N., Yoshizawa A.C. et al. jPOSTrepo: an international standard data repository for proteomes. Nucleic Acids Res. 2017; 45:D1107–D1111. 27899654 10.1093/nar/gkw1080 PMC5210561

[9] Chen T. , Ma J., Liu Y., Chen Z., Xiao N., Lu Y., Fu Y., Yang C., Li M., Wu S. et al. iProX in 2021: connecting proteomics data sharing with big data. Nucleic Acids Res. 2022; 50:D1522–D1527. 34871441 10.1093/nar/gkab1081 PMC8728291

[10] Sharma V. , Eckels J., Schilling B., Ludwig C., Jaffe J.D., MacCoss M.J., MacLean B. Panorama public: a public repository for quantitative data sets processed in skyline. Mol. Cell. Proteomics. 2018; 17:1239–1244. 29487113 10.1074/mcp.RA117.000543 PMC5986241

[11] Deutsch E.W. , Albar J.P., Binz P.A., Eisenacher M., Jones A.R., Mayer G., Omenn G.S., Orchard S., Vizcaino J.A., Hermjakob H. Development of data representation standards by the human proteome organization proteomics standards initiative. J. Am. Med. Inform. Assoc. 2015; 22:495–506. 25726569 10.1093/jamia/ocv001 PMC4457114

[12] Deutsch E.W. , Orchard S., Binz P.A., Bittremieux W., Eisenacher M., Hermjakob H., Kawano S., Lam H., Mayer G., Menschaert G. et al. Proteomics standards initiative: fifteen years of progress and future work. J. Proteome Res. 2017; 16:4288–4298. 28849660 10.1021/acs.jproteome.7b00370 PMC5715286

[13] Perez-Riverol Y. , Xu Q.W., Wang R., Uszkoreit J., Griss J., Sanchez A., Reisinger F., Csordas A., Ternent T., Del-Toro N. et al. PRIDE inspector toolsuite: moving toward a universal visualization tool for proteomics data standard formats and quality assessment of proteomexchange datasets. Mol. Cell. Proteomics. 2016; 15:305–317. 26545397 10.1074/mcp.O115.050229 PMC4762524

[14] Moriya Y. , Kawano S., Okuda S., Watanabe Y., Matsumoto M., Takami T., Kobayashi D., Yamanouchi Y., Araki N., Yoshizawa A.C. et al. The jPOST environment: an integrated proteomics data repository and database. Nucleic Acids Res. 2019; 47:D1218–D1224. 30295851 10.1093/nar/gky899 PMC6324006

[15] Bernal-Llinares M. , Ferrer-Gomez J., Juty N., Goble C., Wimalaratne S.M., Hermjakob H. Identifiers.org: compact identifier services in the cloud. Bioinformatics. 2021; 37:1781–1782. 33031499 10.1093/bioinformatics/btaa864 PMC8289372

[16] Hoyt C.T. , Balk M., Callahan T.J., Domingo-Fernández D., Haendel M.A., Hegde H.B., Himmelstein D.S., Karis K., Kunze J., Lubiana T. et al. Unifying the identification of biomedical entities with the bioregistry. 2022; bioRxiv doi:12 July 2022, preprint: not peer reviewed 10.1101/2022.07.08.499378. PMC9675740 36402838

[17] Martens L. , Chambers M., Sturm M., Kessner D., Levander F., Shofstahl J., Tang W.H., Rompp A., Neumann S., Pizarro A.D. et al. mzML–a community standard for mass spectrometry data. Mol. Cell. Proteomics. 2011; 10:R110 000133. 10.1074/mcp.R110.000133 PMC3013463 20716697

[18] Vizcaino J.A. , Mayer G., Perkins S., Barsnes H., Vaudel M., Perez-Riverol Y., Ternent T., Uszkoreit J., Eisenacher M., Fischer L. et al. The mzIdentML data standard version 1.2, supporting advances in proteome informatics. Mol. Cell. Proteomics. 2017; 16:1275–1285. 28515314 10.1074/mcp.M117.068429 PMC5500760

[19] Griss J. , Jones A.R., Sachsenberg T., Walzer M., Gatto L., Hartler J., Thallinger G.G., Salek R.M., Steinbeck C., Neuhauser N. et al. The mzTab data exchange format: communicating mass-spectrometry-based proteomics and metabolomics experimental results to a wider audience. Mol. Cell. Proteomics. 2014; 13:2765–2775. 24980485 10.1074/mcp.O113.036681 PMC4189001

[20] Pino L.K. , Searle B.C., Bollinger J.G., Nunn B., MacLean B., MacCoss M.J. The skyline ecosystem: informatics for quantitative mass spectrometry proteomics. Mass Spectrom. Rev. 2020; 39:229–244. 28691345 10.1002/mas.21540 PMC5799042

[21] Deutsch E.W. , Perez-Riverol Y., Carver J., Kawano S., Mendoza L., Van Den Bossche T., Gabriels R., Binz P.A., Pullman B., Sun Z. et al. Universal spectrum identifier for mass spectra. Nat. Methods. 2021; 18:768–770. 34183830 10.1038/s41592-021-01184-6 PMC8405201

[22] LeDuc R.D. , Deutsch E.W., Binz P.A., Fellers R.T., Cesnik A.J., Klein J.A., Van Den Bossche T., Gabriels R., Yalavarthi A., Perez-Riverol Y. et al. Proteomics standards initiative's proforma 2.0: unifying the encoding of proteoforms and peptidoforms. J. Proteome Res. 2022; 21:1189–1195. 35290070 10.1021/acs.jproteome.1c00771 PMC7612572

[23] Wang M. , Wang J., Carver J., Pullman B.S., Cha S.W., Bandeira N. Assembling the community-scale discoverable human proteome. Cell Syst. 2018; 7:412–421. 30172843 10.1016/j.cels.2018.08.004 PMC6279426

[24] Dai C. , Fullgrabe A., Pfeuffer J., Solovyeva E.M., Deng J., Moreno P., Kamatchinathan S., Kundu D.J., George N., Fexova S. et al. A proteomics sample metadata representation for multiomics integration and big data analysis. Nat. Commun. 2021; 12:5854. 34615866 10.1038/s41467-021-26111-3 PMC8494749

[25] Choi M. , Carver J., Chiva C., Tzouros M., Huang T., Tsai T.H., Pullman B., Bernhardt O.M., Huttenhain R., Teo G.C. et al. MassIVE.quant: a community resource of quantitative mass spectrometry-based proteomics datasets. Nat. Methods. 2020; 17:981–984. 32929271 10.1038/s41592-020-0955-0 PMC7541731

[26] Na S. , Bandeira N., Paek E. Fast multi-blind modification search through tandem mass spectrometry. Mol. Cell. Proteomics. 2012; 11:M111 010199. 10.1074/mcp.M111.010199 PMC3322561 22186716

[27] van Wijk K.J. , Leppert T., Sun Q., Boguraev S.S., Sun Z., Mendoza L., Deutsch E.W. The arabidopsis peptideatlas: harnessing worldwide proteomics data to create a comprehensive community proteomics resource. Plant Cell. 2021; 33:3421–3453. 34411258 10.1093/plcell/koab211 PMC8566204

[28] Nissa M.U. , Reddy P.J., Pinto N., Sun Z., Ghosh B., Moritz R.L., Goswami M., Srivastava S. The peptideatlas of a widely cultivated fish labeo rohita: a resource for the aquaculture community. Sci. Data. 2022; 9:171. 35418183 10.1038/s41597-022-01259-9 PMC9008064

[29] Reales-Calderon J.A. , Sun Z., Mascaraque V., Perez-Navarro E., Vialas V., Deutsch E.W., Moritz R.L., Gil C., Martinez J.L., Molero G. A wide-ranging pseudomonas aeruginosa peptideatlas build: a useful proteomic resource for a versatile pathogen. J. Proteomics. 2021; 239:104192. 33757883 10.1016/j.jprot.2021.104192 PMC8668395

[30] Midha M.K. , Campbell D.S., Kapil C., Kusebauch U., Hoopmann M.R., Bader S.L., Moritz R.L. DIALib-QC an assessment tool for spectral libraries in data-independent acquisition proteomics. Nat. Commun. 2020; 11:5251. 33067471 10.1038/s41467-020-18901-y PMC7567827

[31] Adhikari S. , Nice E.C., Deutsch E.W., Lane L., Omenn G.S., Pennington S.R., Paik Y.K., Overall C.M., Corrales F.J., Cristea I.M. et al. A high-stringency blueprint of the human proteome. Nat. Commun. 2020; 11:5301. 33067450 10.1038/s41467-020-19045-9 PMC7568584

[32] UniProt C. UniProt: the universal protein knowledgebase in 2021. Nucleic Acids Res. 2021; 49:D480–D489. 33237286 10.1093/nar/gkaa1100 PMC7778908

[33] Zahn-Zabal M. , Michel P.A., Gateau A., Nikitin F., Schaeffer M., Audot E., Gaudet P., Duek P.D., Teixeira D., Rech de Laval V. et al. The neXtProt knowledgebase in 2020: data, tools and usability improvements. Nucleic Acids Res. 2020; 48:D328–D334. 31724716 10.1093/nar/gkz995 PMC7145669

[34] Ramsbottom K.A. , Prakash A., Riverol Y.P., Camacho O.M., Martin M.J., Vizcaino J.A., Deutsch E.W., Jones A.R. Method for independent estimation of the false localization rate for phosphoproteomics. J. Proteome Res. 2022; 21:1603–1615. 35640880 10.1021/acs.jproteome.1c00827 PMC9251759

[35] Ramasamy P. , Turan D., Tichshenko N., Hulstaert N., Vandermarliere E., Vranken W., Martens L. Scop3P: a comprehensive resource of human phosphosites within their full context. J. Proteome Res. 2020; 19:3478–3486. 32508104 10.1021/acs.jproteome.0c00306

[36] Craig R. , Cortens J.P., Beavis R.C. Open source system for analyzing, validating, and storing protein identification data. J. Proteome Res. 2004; 3:1234–1242. 15595733 10.1021/pr049882h

[37] Cunningham F. , Allen J.E., Allen J., Alvarez-Jarreta J., Amode M.R., Armean I.M., Austine-Orimoloye O., Azov A.G., Barnes I., Bennett R. et al. Ensembl 2022. Nucleic Acids Res. 2022; 50:D988–D995. 34791404 10.1093/nar/gkab1049 PMC8728283

[38] Lee B.T. , Barber G.P., Benet-Pages A., Casper J., Clawson H., Diekhans M., Fischer C., Gonzalez J.N., Hinrichs A.S., Lee C.M. et al. The UCSC genome browser database: 2022 update. Nucleic Acids Res. 2022; 50:D1115–D1122. 34718705 10.1093/nar/gkab959 PMC8728131

[39] Mudge J.M. , Ruiz-Orera J., Prensner J.R., Brunet M.A., Calvet F., Jungreis I., Gonzalez J.M., Magrane M., Martinez T.F., Schulz J.F. et al. Standardized annotation of translated open reading frames. Nat. Biotechnol. 2022; 40:994–999. 35831657 10.1038/s41587-022-01369-0 PMC9757701

[40] Volders P.J. , Anckaert J., Verheggen K., Nuytens J., Martens L., Mestdagh P., Vandesompele J. LNCipedia 5: towards a reference set of human long non-coding RNAs. Nucleic Acids Res. 2019; 47:D135–D139. 30371849 10.1093/nar/gky1031 PMC6323963

[41] Olexiouk V. , Crappe J., Verbruggen S., Verhegen K., Martens L., Menschaert G. sORFs.org: a repository of small ORFs identified by ribosome profiling. Nucleic Acids Res. 2016; 44:D324–D329. 26527729 10.1093/nar/gkv1175 PMC4702841

[42] Brunet M.A. , Lucier J.F., Levesque M., Leblanc S., Jacques J.F., Al-Saedi H.R.H., Guilloy N., Grenier F., Avino M., Fournier I. et al. OpenProt 2021: deeper functional annotation of the coding potential of eukaryotic genomes. Nucleic Acids Res. 2021; 49:D380–D388. 33179748 10.1093/nar/gkaa1036 PMC7779043

[43] Mitchell A.L. , Almeida A., Beracochea M., Boland M., Burgin J., Cochrane G., Crusoe M.R., Kale V., Potter S.C., Richardson L.J. et al. MGnify: the microbiome analysis resource in 2020. Nucleic Acids Res. 2020; 48:D570–D578. 31696235 10.1093/nar/gkz1035 PMC7145632

[44] Shao W. , Pedrioli P.G.A., Wolski W., Scurtescu C., Schmid E., Vizcaino J.A., Courcelles M., Schuster H., Kowalewski D., Marino F. et al. The SysteMHC atlas project. Nucleic Acids Res. 2018; 46:D1237–D1247. 28985418 10.1093/nar/gkx664 PMC5753376

[45] Moreno P. , Fexova S., George N., Manning J.R., Miao Z., Mohammed S., Munoz-Pomer A., Fullgrabe A., Bi Y., Bush N. et al. Expression atlas update: gene and protein expression in multiple species. Nucleic Acids Res. 2022; 50:D129–D140. 34850121 10.1093/nar/gkab1030 PMC8728300

[46] Jarnuczak A.F. , Najgebauer H., Barzine M., Kundu D.J., Ghavidel F., Perez-Riverol Y., Papatheodorou I., Brazma A., Vizcaino J.A. An integrated landscape of protein expression in human cancer. Sci Data. 2021; 8:115. 33893311 10.1038/s41597-021-00890-2 PMC8065022

[47] Prakash A. , García-Seisdedos D., Wang S., Kundu D.J., Collins A., George N., Moreno P., Papatheodorou I., Jones A.R., Vizcaíno J.A. An integrated view of baseline protein expression in human tissues. 2022; bioRxiv doi:21 October 2022, preprint: not peer reviewed 10.1101/2021.09.10.459811. PMC9990129 36577097

[48] Wang S. , Garcia-Seisdedos D., Prakash A., Kundu D.J., Collins A., George N., Fexova S., Moreno P., Papatheodorou I., Jones A.R. et al. Integrated view and comparative analysis of baseline protein expression in mouse and rat tissues. PLoS Comput. Biol. 2022; 18:e1010174. 35714157 10.1371/journal.pcbi.1010174 PMC9246241

[49] Walzer M. , Garcia-Seisdedos D., Prakash A., Brack P., Crowther P., Graham R.L., George N., Mohammed S., Moreno P., Papatheodorou I. et al. Implementing the reuse of public DIA proteomics datasets: from the PRIDE database to expression atlas. Sci. Data. 2022; 9:335. 35701420 10.1038/s41597-022-01380-9 PMC9197839

[50] Lautenbacher L. , Samaras P., Muller J., Grafberger A., Shraideh M., Rank J., Fuchs S.T., Schmidt T.K., The M., Dallago C. et al. ProteomicsDB: toward a FAIR open-source resource for life-science research. Nucleic Acids Res. 2022; 50:D1541–D1552. 34791421 10.1093/nar/gkab1026 PMC8728203

[51] Shao X. , Taha I.N., Clauser K.R., Gao Y.T., Naba A. MatrisomeDB: the ECM-protein knowledge database. Nucleic Acids Res. 2020; 48:D1136–D1144. 31586405 10.1093/nar/gkz849 PMC6943062

[52] Kustatscher G. , Grabowski P., Schrader T.A., Passmore J.B., Schrader M., Rappsilber J. Co-regulation map of the human proteome enables identification of protein functions. Nat. Biotechnol. 2019; 37:1361–1371. 31690884 10.1038/s41587-019-0298-5 PMC6901355

[53] Mann M. , Kumar C., Zeng W.F., Strauss M.T. Artificial intelligence for proteomics and biomarker discovery. Cell Syst. 2021; 12:759–770. 34411543 10.1016/j.cels.2021.06.006

[54] Ochoa D. , Jarnuczak A.F., Vieitez C., Gehre M., Soucheray M., Mateus A., Kleefeldt A.A., Hill A., Garcia-Alonso L., Stein F. et al. The functional landscape of the human phosphoproteome. Nat. Biotechnol. 2020; 38:365–373. 31819260 10.1038/s41587-019-0344-3 PMC7100915

[55] Perez-Riverol Y. , Zorin A., Dass G., Vu M.T., Xu P., Glont M., Vizcaino J.A., Jarnuczak A.F., Petryszak R., Ping P. et al. Quantifying the impact of public omics data. Nat. Commun. 2019; 10:3512. 31383865 10.1038/s41467-019-11461-w PMC6683138

[56] Bandeira N. , Deutsch E.W., Kohlbacher O., Martens L., Vizcaino J.A. Data management of sensitive human proteomics data: current practices, recommendations and perspectives for the future. Mol. Cell. Proteomics. 2021; 20:100071. 33711481 10.1016/j.mcpro.2021.100071 PMC8056256

[57] Freeberg M.A. , Fromont L.A., D’Altri T., Romero A.F., Ciges J.I., Jene A., Kerry G., Moldes M., Ariosa R., Bahena S. et al. The european Genome-phenome archive in 2021. Nucleic Acids Res. 2022; 50:D980–D987. 34791407 10.1093/nar/gkab1059 PMC8728218

[58] Tryka K.A. , Hao L., Sturcke A., Jin Y., Wang Z.Y., Ziyabari L., Lee M., Popova N., Sharopova N., Kimura M. et al. NCBI’s database of genotypes and phenotypes: dbGaP. Nucleic Acids Res. 2014; 42:D975–D979. 24297256 10.1093/nar/gkt1211 PMC3965052

[59] Okido T. , Kodama Y., Mashima J., Kosuge T., Fujisawa T., Ogasawara O. DNA data bank of japan (DDBJ) update report 2021. Nucleic Acids Res. 2022; 50:D102–D105. 34751405 10.1093/nar/gkab995 PMC8689959

[60] Keane T.M. , O’Donovan C., Vizcaino J.A. The growing need for controlled data access models in clinical proteomics and metabolomics. Nat. Commun. 2021; 12:5787. 34599180 10.1038/s41467-021-26110-4 PMC8486822

[61] Armstrong D.R. , Berrisford J.M., Conroy M.J., Gutmanas A., Anyango S., Choudhary P., Clark A.R., Dana J.M., Deshpande M., Dunlop R. et al. PDBe: improved findability of macromolecular structure data in the PDB. Nucleic Acids Res. 2020; 48:D335–D343. 31691821 10.1093/nar/gkz990 PMC7145656

[62] Jones A.R. , Deutsch E.W., Vizcaino J.A. Is DIA proteomics data FAIR? Current data sharing practices, available bioinformatics infrastructure and recommendations for the future. Proteomics. 2022; e2200014. 36074795 10.1002/pmic.202200014 PMC10155627

