# Supplementary material for: The ProteomeXchange consortium at 10 years: 2023 update
Source: Nucleic Acids Res. 2022 Nov 12;51(D1):D1539–48. doi: 10.1093/nar/gkac1040 (PMC9825490; doi:10.1093/nar/gkac1040)
Supplement: gkac1040_Supplemental_File [file gkac1040_supplemental_file.docx]

**Supplementary Table 1.** Average monthly downloads statistics for the different ProteomeXchange resources during 2019, 2020 and 2021. The download protocols covered are: FTP (for PRIDE, MassIVE, PeptideAtlas, jPOST), Aspera (for PRIDE and iProX), and HTPP and HTPPS (for jPOST and iProX). Custom log analysis was used for PeptideAtlas, PRIDE, MassIVE and Panorama Public. AWStats was used in the case of jPOST and iProX. N/A: Not available.

| **Average monthly downloads** | **2019** | **2020** | **2021** |
| --- | --- | --- | --- |
| *Hits / Requests per month* | PRIDE: 233,474  MassIVE: 1,588,777  jPOST: 20,634  iProX: 176,671  Panorama: N/A  PeptideAtlas: 1,035  ProteomeCentral: N/A  TOTAL: 2,020,591 | PRIDE: 15,467,773  MassIVE: 1,715,986  jPOST: 47,812  iProX: 341,924  Panorama: N/A  PeptideAtlas: 1,897  ProteomeCentral: N/A  TOTAL: 17,575,392 | PRIDE: 1,035,093  MassIVE: 8,036,093  jPOST: 47,990  iProX: 618,903  Panorama: N/A  PeptideAtlas: 2,871  ProteomeCentral: N/A  TOTAL: 9,740,950 |
| *Unique IP addresses / Hosts per month* | PRIDE: 1,355  MassIVE: 1,543  jPOST: 12  iProX: 1,736  Panorama: N/A  PeptideAtlas: N/A  ProteomeCentral: N/A  TOTAL: 4,646 | PRIDE: 19,720  MassIVE: 4,595  jPOST: 191  iProX: 1,439  Panorama: N/A  PeptideAtlas: N/A  ProteomeCentral: N/A  TOTAL: 25,945 | PRIDE: 7,346  MassIVE: 15,435  jPOST: 207  iProX: 7,602  Panorama: N/A  PeptideAtlas: N/A  ProteomeCentral: N/A  TOTAL: 30,590 |
| *Data transfer per month (in GBs)* | PRIDE: 81,890  MassIVE: 54,356  jPOST: 66.81  iProX: 11,173  Panorama: N/A  PeptideAtlas: N/A  ProteomeCentral: N/A  TOTAL: 147,486 | PRIDE: 144,278  MassIVE: 48,215  jPOST: 226.68  iProX: 28,565  Panorama: N/A  PeptideAtlas: N/A  ProteomeCentral: N/A  TOTAL: 221,285 | PRIDE: 78,115  MassIVE: 95,253  jPOST: 875.11  iProX: 36,312  Panorama: N/A  PeptideAtlas: N/A  ProteomeCentral: N/A  TOTAL: 210,555 |
